# A comprehensive investigation of metagenome assembly by linked-read sequencing

**DOI:** 10.1186/s40168-020-00929-3

**Published:** 2020-11-11

**Authors:** Lu Zhang, Xiaodong Fang, Herui Liao, Zhenmiao Zhang, Xin Zhou, Lijuan Han, Yang Chen, Qinwei Qiu, Shuai Cheng Li

**Affiliations:** 1 Department of Computer Science, Hong Kong Baptist University, Kowloon Tong, Hong Kong China; 2 Department of Computer Science, City University of Hong Kong, Kowloon Tong, Hong Kong China; 3 KMBGI GeneTech Co., Ltd., Shenzhen, Guangdong China; 4 Department of Electronic Engineering, City University of Hong Kong, Hong Kong, China; 5 Department of Computer Science, Stanford University, Stanford, CA USA; 6 State Key Laboratory of Dampness Syndrome of Chinese Medicine, The Second Affiliated Hospital of Guangzhou University of Chinese Medicine, Guangzhou, Guangdong China

**Keywords:** Metagenome assembly, Linked-reads, Short-reads, PacBio CCS long-reads, Parameter space

## Abstract

**Background:**

The human microbiota are complex systems with important roles in our physiological activities and diseases. Sequencing the microbial genomes in the microbiota can help in our interpretation of their activities. The vast majority of the microbes in the microbiota cannot be isolated for individual sequencing. Current metagenomics practices use short-read sequencing to simultaneously sequence a mixture of microbial genomes. However, these results are in ambiguity during genome assembly, leading to unsatisfactory microbial genome completeness and contig continuity. Linked-read sequencing is able to remove some of these ambiguities by attaching the same barcode to the reads from a long DNA fragment (10–100 kb), thus improving metagenome assembly. However, it is not clear how the choices for several parameters in the use of linked-read sequencing affect the assembly quality.

**Results:**

We first examined the effects of *read depth* (*C*) on metagenome assembly from linked-reads in simulated data and a mock community. The results showed that *C* positively correlated with the length of assembled sequences but had little effect on their qualities. The latter observation was corroborated by tests using real data from the human gut microbiome, where *C* demonstrated minor impact on the sequence quality as well as on the proportion of bins annotated as draft genomes. On the other hand, metagenome assembly quality was susceptible to *read depth per fragment* (*C*_*R*_) and *DNA fragment physical depth* (*C*_*F*_). For the same *C*, deeper *C*_*R*_ resulted in more draft genomes while deeper *C*_*F*_ improved the quality of the draft genomes. We also found that *average fragment length* (*μ*_*FL*_) had marginal effect on assemblies, while *fragments per partition* (*N*_*F/P*_) impacted the off-target reads involved in local assembly, namely, lower *N*_*F/P*_ values would lead to better assemblies by reducing the ambiguities of the off-target reads. In general, the use of linked-reads improved the assembly for contig N50 when compared to Illumina short-reads, but not when compared to PacBio CCS (circular consensus sequencing) long-reads.

**Conclusions:**

We investigated the influence of linked-read sequencing parameters on metagenome assembly comprehensively. While the quality of genome assembly from linked-reads cannot rival that from PacBio CCS long-reads, the case for using linked-read sequencing remains persuasive due to its low cost and high base-quality. Our study revealed that the probable best practice in using linked-reads for metagenome assembly was to merge the linked-reads from multiple libraries, where each had sufficient *C*_*R*_ but a smaller amount of input DNA.

Video Abstract

## Background

The human microbiota are complex systems that contribute to a large part of human physiological activities and diseases. Knowing the genomic sequences of the microbiota content allows us to study its functions. However, microbial genome sequences are difficult to obtain. While a few microbes can survive isolation and be cultured in vitro for sequencing, the remaining microbial content remains as “microbial dark matter”. Alternatively, there have been attempts to use computational means to reconstruct the microbial genomes from a mixture of short-reads sequenced from them. However, such metagenome assembly faces the difficulties of having repetitive sequences of both intra- and inter-species origin, horizontal gene transfers, and mobilization events [1], complicated by uneven abundance of microbes in the sample.

Current algorithms such as IDBA-UD [2], MEGAHIT [3], and MetaSPAdes [4] make use of read depth and fragment insert size constraint to unravel the repetitive sequences and estimate microbial abundance. However, their reliability is affected by the low continuity of short-read assembly. Long-read sequencing has been used to attempt to mitigate these problems, e.g., Nicholls et al. [5] and Sevim et al. [6]. In particular, Moss et al*.* [7] optimized the long-read library preparation protocol of nanopore sequencing and produced more complete bacterial genomes. However, the application of long-read sequencing in practical application remains costly (the “Discussion” section).

Alternative sequencing platforms that provide long-range sequence information for metagenomics are available in the form of Illumina Truseq Synthetic Long Reads (SLR) and linked-reads. SLR arranges long DNA fragments into 384 well plates, which are further amplified and pooled sequenced with sufficiently deep sequencing depth (~ 50X per fragment), thus allowing long fragments to be assembled individually [8, 9]. Linked-reads are short-reads where reads from the same fragment are marked with the same barcode. The 10x linked-read microfluidic system assigns long DNA fragments into around 1 million partitions, where each fragment is sequenced with a shallow depth (0.1X–0.4X). A method for linked-read metagenome assembly, Athena-meta [10], bridges the gaps between contigs by local assembly on co-barcoded reads and outperformed the methods for short-reads and SLR in assembling human gut and environmental microbiome.

**Fig. 1:**
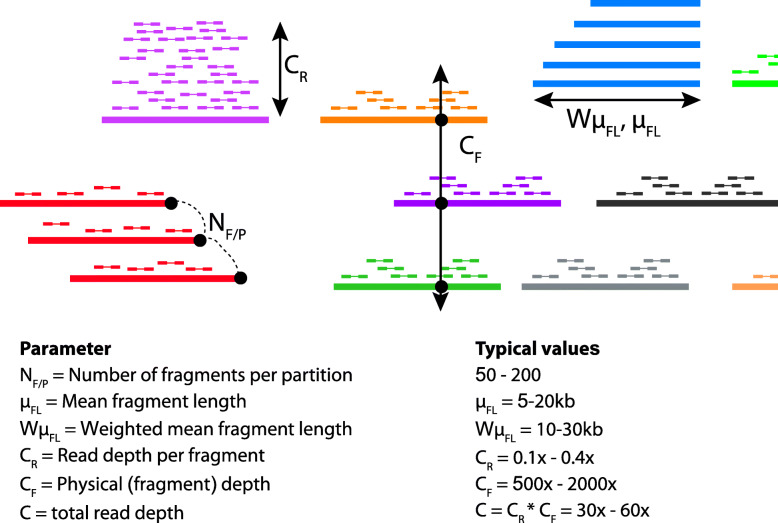
Parameters of linked-read sequencing to be investigated

There are four key parameters in linked-read sequencing which may impact metagenome assembly [11] (Fig. 1): (i) *C*_*R*_, *average depth of short-reads per fragment*; (ii) *C*_*F*_: *average physical depth of the genome by long DNA fragments*; (iii) *N*_*F/P*_, *number of fragments per partition*; (iv) *Fragment length distribution*, which is specified using two parameters, namely, *μ*_*FL*_—*average unweighted DNA fragment length* and W*μ*_*FL*_—*length-weighted average of DNA fragment length*. Several of these parameters are interdependent. For example, a greater amount of input DNA increases both *C*_*F*_ and *N*_*F/P*_ and decrease *C*_*R*_; and the absolute values of *C*_*F*_ and *C*_*R*_ are set by how much total read coverage (*C*) is generated because *C*_*R*_ × *C*_*F*_ = *C*. In a previous study, we investigated the effects of these parameters on human diploid assembly [11].

The present study evaluates these parameters with respect to their impact on metagenome assembly. We used three sets of linked-reads, one from simulation, one from a mock community, and another from a real human gut microbiome sample. The simulated data consists of twenty datasets (Table S1) generated by an improved LRTK-SIM [11] that enables to deal with microbial samples with uneven abundance for this study (the “Methods” section). The mock community (ATCC MSA-1003) is a pool of 20 strains with staggered abundance, while the human gut microbiome is from a healthy Chinese stool sample. Because of an absence of ground truth to evaluate human gut microbiome, we annotated contig bins as draft genomes and assigned them to the corresponding taxonomic classification (the “Methods” section).

Our results show that deeper *C* resulted in more assembled sequences and enabled better genomic coverage, but it was irrelevant to the assembly quality. *C* was not a dominating factor for contig continuity, which could be influenced more by genome characteristics. We further found *C*_*R*_ to affect the number of draft genomes and that *C*_*F*_ was associated with assembly quality. The *μ*_*FL*_ had marginal effect on assemblies, and lower *N*_*F/P*_ values would lead to better assemblies by reducing the ambiguities of off-target reads. Compared to Illumina short-reads, 10x linked-reads significantly improved the metagenome assembly in both contig continuity and genome completeness.

## Results

Three sets of linked-reads are used. The first is simulated from the MBARC-26 [6] community (Table S1 and S2), and the twenty simulated datasets are annotated as ${C}_F^{-}$, ${C}_R^{-}$,${\mu}_{FL}^{-}$, and ${N}_{F/P}^{-}$(where superscript “-” represents the actual values of corresponding parameters, Table S1). The second and third sets are sequenced from a mock community of 20 strains (one lane reads from Illumina XTen, 108.7 GB, Table S3) and a human gut microbiome (two lane reads from Illumina XTen, 208.97 GB; the “Methods” section and Supplementary Note) followed by reads subsampling to match the expected parameter values. The microbial complexity in the human gut microbiome was evaluated by aligning linked-reads to the reference sequences from human microbiome project [12] (Supplementary Note). To obtain the datasets of different *C*_*R*_ and *C*_*F*_, we subsampled short-reads (MS*C*_*R-*_) and long DNA fragments (MS*C*_*F-*_) of the mock community (the “Methods” section), where value of subscript “-” represents the reciprocal of sequenced lanes—for example, MS*C*_*R4*_/MSC_*F4*_ means quarter lane reads were subsampled. Since the composition of the human gut microbiome is unknown, S*C*_*R-*_ and S*C*_*F-*_ (where subscript “-” represents the reciprocal of sequenced lanes) were generated by subsampling short-reads and barcodes instead. To avoid confusion, we used MS*C*_*1*_ and S*C*_*all*_ to denote total one lane and two lanes linked-reads from the mock community and human gut microbiome, respectively.

According to microbial relative abundance, the microbes were classified into low- (*L*_*sim*_), medium- (*M*_*sim*_), and high-abundance (*H*_*sim*_) in the simulated data (Table S2); and classified into low- (*L*_*mock*_), medium- (*M*_*mock*_), high- (*H*_*mock*_), and ultrahigh-abundance (*UH*_*mock*_) in the mock community (Table S3). The contigs from the simulation and mock community were evaluated using two reference-based metrics (total aligned length and genomic coverage) and two measures for contig continuity (contig NG50 and NGA50). For human gut microbiome data, we annotated the contig bins as draft genomes and classified them into high-, medium-, and low-quality [13] (the “Methods” section). The number and quality of annotated draft genomes and contig N50 were used to evaluate the assemblies.

### The influence of total read depth *C*

**Fig. 2:**
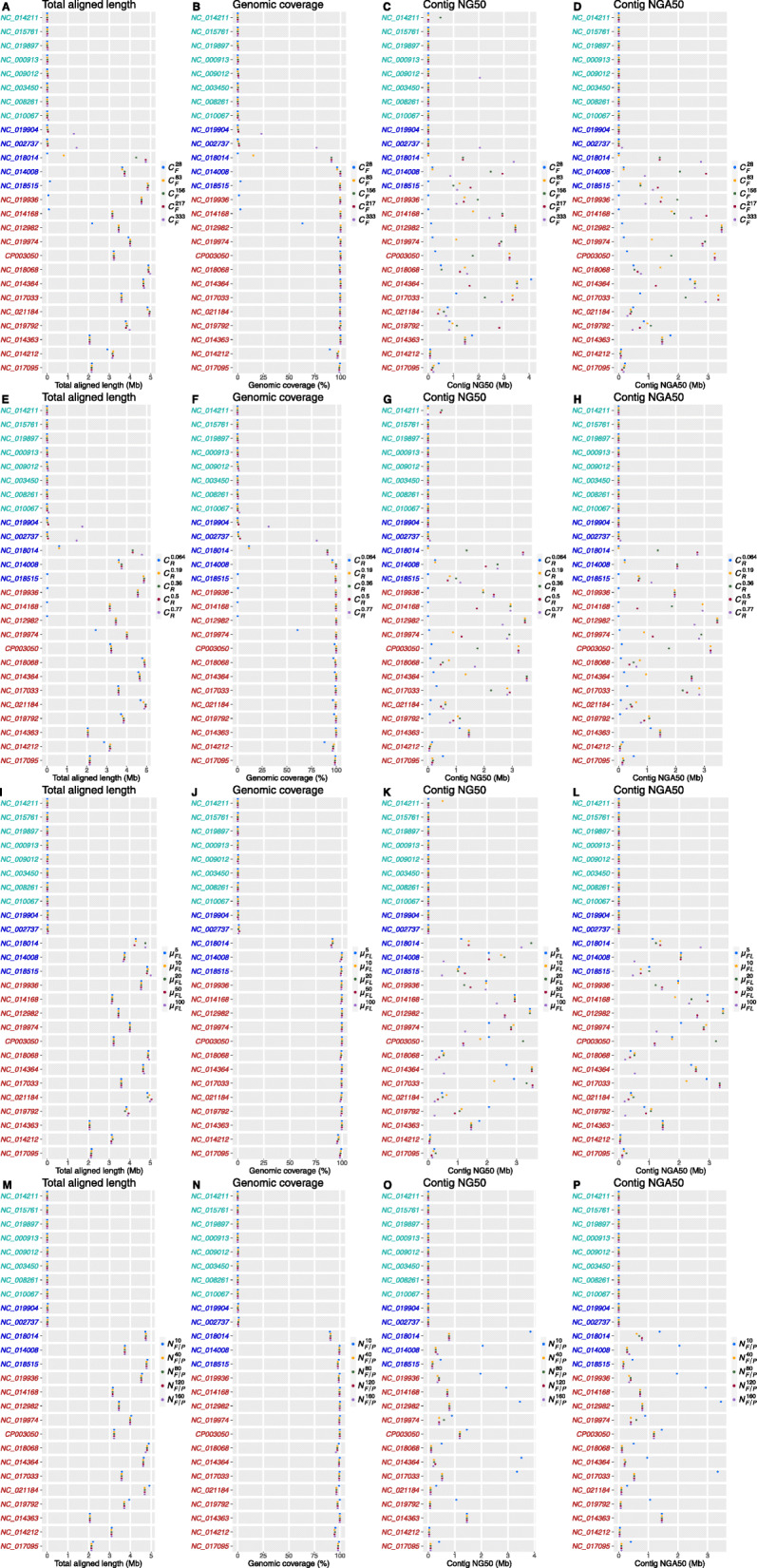
Trends of total assembly length, genomic coverage, contig NG50, and NGA50 by subsampling *C*_*R*_ (**a**–**d**), *C*_*F*_ (**e**–**h**), *μ*_*FL*_ (**i**–**l**), and *N*_*F/P*_ (**m**–**p**) in simulated data. Microbes in cyan, blue, and red represent *L*_*sim*_, *M*_*sim*_*,* and *H*_*sim*_ species, respectively

*C* has little effect on both total aligned length and genomic coverage for *L*_*sim*_ and *H*_*sim*_ microbes in the simulated data. For *M*_*sim*_ microbes, their abundance correlates positively with total aligned length and genomic coverage, indicating that a low abundance could reduce assembly completeness even when *C* is high (Fig. 2a, b, e, and f).

Similarly, we fail to observe any clear trend between NG50 (or NGA50) and *C.* Two microbes with the deepest *C*, NC_014212 and NC_017095 (with the highest abundance), were assembled into fragmented contigs (Fig. 2c and g), suggesting that *C* was not a dominating factor for contig continuity; which is also seen in ${C}_F^{333}$ and ${C}_R^{0.77}$, which have deeper *C* (*C* = 120X) than the other configurations. They achieved the largest total aligned length and genomic coverage, but their contig NG50 and NGA50 fluctuated and were not always the best. For example, NC_019904 and NC_002737, which have the lowest abundance among *M*_*sim*_ microbes, yielded the largest total aligned length in ${C}_F^{333}$ (NC_019904, 1.29 Mb; NC_002737, 1.42 Mb; Fig. 2a) and ${C}_R^{0.77}$ (NC_019904, 1.77 Mb, NC_002737, 1.49 Mb; Fig. 2e). ${C}_R^{0.77}$ assembled fragmented contigs for both of the microbes on NG50 (NC_019904 < 500 bp, NC_002737 = 64.14 kb; Fig. 2g) and NGA50 (NC_019904 < 500 bp, NC_002737 = 62.47 kb; Fig. 2h). Although ${C}_F^{333}$ produced better NG50 on NC_002737 (NG50, 2.04 Mb, Fig. 2c), misassemblies were dispersed in its contigs (NGA50, 109.43 kb; Fig. 2d).

**Fig. 3:**
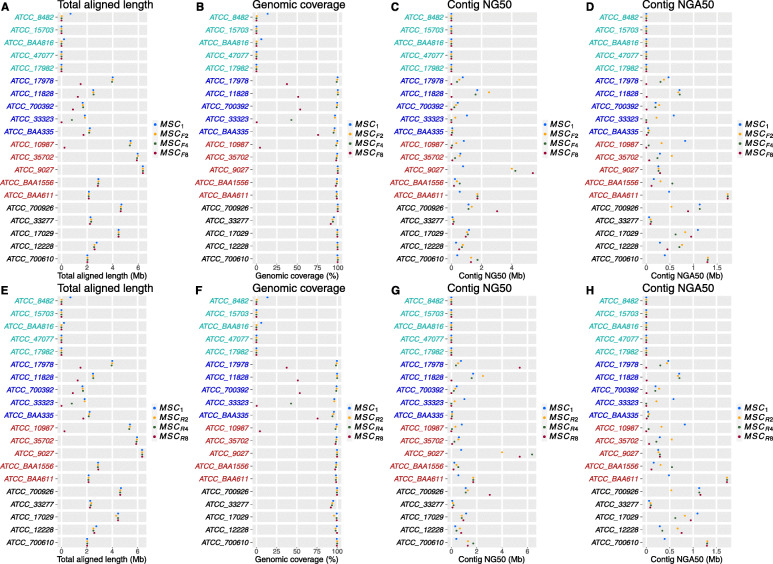
Trends of total assembly length, genomic coverage, contig NG50, and NGA50 by subsampling *C*_*R*_ (**a**–**d**) and *C*_*F*_ (**e–h**) for the mock community. Microbes in cyan, blue, red, and black represent *L*_*mock*_, *M*_*mock*_ and *H*_*mock*_, and *UH*_*mock*_ species, respectively

The results for the mock community are consistent with those from the simulated data. The total aligned length and genomic coverage were fairly stable for *L*_*mock*_, *H*_*mock*_, and *UH*_*mock*_ microbes regardless of the value of *C* (Fig. 3a, b, e, and f). For *M*_*mock*_ microbes, the contigs from MS*C*_*F8*_/MS*C*_*R8*_ covered the reference genomes poorly due to the insufficient read depth. A quarter lane reads (MS*C*_*F4*_/MS*C*_*R4*_) appeared to suffice for the read depth, achieving around full genomic coverage for all *M*_*mock*_ microbes, except for ATCC_33323, which required a quarter lane reads more. No consistent trend could be observed for NG50 and NGA50; a quarter lane reads was necessary to generate contigs with non-zero NGA50 for *M*_*mock*_ microbes.

**Fig. 4:**
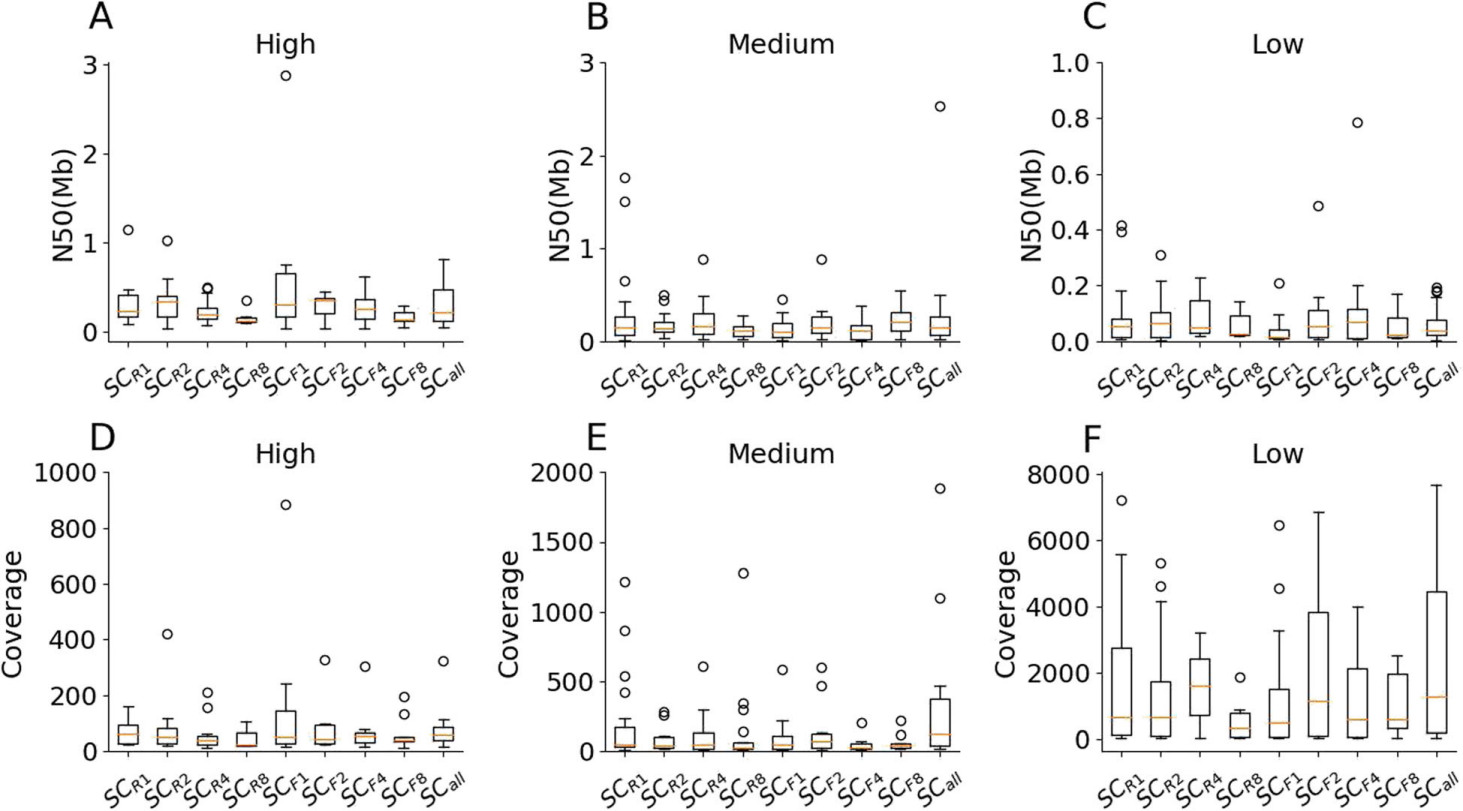
Contig N50 and read depth for high-, medium-, and low-quality bins

In the results with human gut microbiome, deeper *C* extends the assembly length but has no impact on the assembly quality. After binning contigs and classifying the bins into draft genomes (the “Methods” section), S*C*_*all*_ produced the largest number of bins (148) and the longest assembly length (399.41 Mb). These statistics were reduced along subsampling reads progressively (Table 1). The proportions of bins annotated as draft genomes were reduced by increasing *C* (S*C*_*R8*_ 77.78%, S*C*_*R4*_ 69.39%, S*C*_*R2*_ 63.75%, S*C*_*R1*_ 65.38%, S*C*_*all*_ 54.05%; S*C*_*F8*_ 68.75%, S*C*_*F4*_ 60.94%, S*C*_*F2*_ 59.55%, S*C*_*F1*_ 58.16%, S*C*_*all*_ 54.05%). *C* negatively correlates with bin average contamination (S*C*_*all*_ 14.40%, S*C*_*R2*_ 10.46%, S*C*_*R4*_ 9.08%, S*C*_*R8*_ 8.94%; Table S4 and Figure S1). We annotated the draft genomes as genus or species (> 60% confidence) based on their *k*-mer similarities with known microbial genomes (the “Methods” section). Most of the taxonomical classifications were observed by at least two parameter configurations, although some were unique to only one (Figure S2). Considering the qualities of annotated draft genomes, *C* demonstrated a positive correlation with the number of medium- and low-quality bins (Table 1); S*C*_*R4*_ has the most high-quality bins and the largest average bin completeness (73.3%) compared to the other configurations (Table 1 and Table S4). The N50s of high-quality bins are significantly greater than medium- (*p* value = 0.01) and low-quality (*p* value = 5.3E−9) bins, suggesting that bin quality (determined by completeness and contamination) is highly correlated with contig continuities (Fig. 4a–c). Interestingly, high-quality bins required read coverage of at least 50X (S*C*_*all*_ = 85.81X; S*C*_*F1*_ = 132.71X; S*C*_*F2*_ = 111.11X; S*C*_*F4*_ = 96.43X; S*C*_*F8*_ = 64.67X; S*C*_*R1*_ = 75.66X; S*C*_*R2*_ = 151.55X; S*C*_*R4*_ = 63.42X; S*C*_*R8*_ = 53.08X), suggesting that the low abundance microbes were not assembled into high-quality genomes. Nevertheless, the contigs with extremely high depth may come from repetitive sequences and reduce the qualities of bins they belong to (Fig. 4d–f; *C* (high) = 81.4X; *C* (medium) = 140.1X; *C* (low) = 1636.5X).

**Table 1:** Summary of the assemblies for subsampled linked-reads from human gut microbiome and Illumina short-reads

Configurations	No. of bins	Total length (Mb)	High (%)	Medium (%)	Low (%)	Others (%)
**S*C***_**all**_	**148**	**399.41**	9 (6.08%)	23 (15.54)	**48 (32.43)**	**68 (45.95)**
**S*C***_***R1***_	104	290.49	10 (9.62)	**30 (28.85)**	28 (26.92)	36 (34.62)
**S*C***_***R2***_	80	225.73	11 (13.75)	15 (18.75)	25 (31.25)	29 (36.25)
**S*C***_***R4***_	49	159.40	**15** (**30.61**)	9 (18.37)	10 (20.41)	15 (30.61)
**S*C***_***R8***_	36	115.96	6 (16.67)	16 (44.44)	6 (16.67)	8 (22.22)
**S*C***_***F1***_	98	305.24	14 (14.29)	20 (20.41)	23 (23.47)	41(41.84)
**S*C***_***F2***_	89	244.55	7 (7.87)	16 (17.98)	30 (33.71)	36 (40.45)
**S*C***_***F4***_	64	188.90	7 (10.94)	13 (20.31)	19 (29.69)	25 (39.06)
**S*C***_***F8***_	48	152.65	9 (18.75)	10 (20.83)	14 (29.17)	15 (31.25)
**ILLU**	53	145.50	0 (0)	16 (30.19)	16 (30.19)	21 (39.62)

*ILLU* assembly from Illumina short-reads

### The tradeoffs between *C*_*R*_ and *C*_*F*_

There are tradeoffs between *C*_*R*_ and *C*_*F*_ in maintaining the same *C*. Because the product of PCR amplification per partition can generate around 500 Mb short-reads, loading DNA with greater density (deeper *C*_*F*_) results in more fragments per partition and fewer reads sequenced for each fragment (shallower *C*_*R*_). For *M*_*sim*_ and *H*_*sim*_ species in the simulated data, we found that increasing *C*_*R*_ is more effective than increasing *C*_*F*_ when *C* is around 10X, and they are comparably effective when *C* is beyond 30X (Fig. 2a, b, e, and f). As a rule, deep *C*_*R*_ is more pressing to reconstruct DNA fragment if *C* is low. For the examples of ${C}_F^{28}$ and ${C}_R^{0.064}$ (*C* = 10x), ${C}_F^{28}$ (*C*_*R*_ = 0.36X) was significantly better than ${C}_R^{0.064}$ (*C*_*R*_ = 0.064X) in total aligned length (${C}_F^{28}:{C}_R^{0.064}$ = 2.17 Mb: < 500 bp) and genomic coverage (${C}_F^{28}:{C}_R^{0.064}$ = 62.93%: < 1%) for NC_012982. ${C}_F^{28}$ generated more continuous contigs than ${C}_R^{0.064}$ for the five *H*_*sim*_ species, NC_018068, NC_014364, NC_017033, NC021184, and NC_019792, (Fig. 2 c, d, g and h). In the mock community, MS*C*_*F-*_ and MS*C*_*R-*_ produced comparable assemblies when *C* was kept constant.

In human gut microbiome, S*C*_*F-*_ generated more assembled sequences than S*C*_*R-*_(S*C*_*F1*_:S*C*_*R1*_ = 305.24 Mb:290.49 MB; S*C*_*F2*_:S*C*_*R2*_ = 244.55 Mb:225.73 Mb; S*C*_*F4*_:S*C*_*R4*_ = 188.90 Mb:159.40 Mb; S*C*_*F8*_:S*C*_*R8*_ = 152.65 Mb:115.96 Mb, Table 1), but had higher average bin contamination (S*C*_*F1*_:S*C*_*R1*_ = 14.04%:12.10%; S*C*_*F2*_:S*C*_*R2*_ = 14.80%:10.46%; S*C*_*F4*_:S*C*_*R4*_ = 12.66%:9.08%; S*C*_*F8*_:S*C*_*R8*_ = 12.17%:8.95%) and worse contig N50 (S*C*_*F1*_:S*C*_*R1*_ = 137.66 kb:168.67 kb; S*C*_*F2*_:S*C*_*R2*_ = 127.58 kb:151.49 kb; S*C*_*F4*_:S*C*_*R4*_ = 136.40 kb:181.46 kb; S*C*_*F8*_:S*C*_*R8*_ = 115.29 kb:118.0 kb). These observations suggest that deeper *C*_*R*_ would result in more assembled sequences, while deeper *C*_*F*_ would help in improving assembly quality.

### DNA fragment length and metagenome assembly

DNA long fragment information is critical for linked-read assembly, as it can help in spanning the gaps between contig breaks that are due to genome variations and repetitive sequences. On the other hand, it may lead to the loss of barcode specificity in disentangling short tandem repeats if the fragments are exceedingly long.

In practice, it is difficult to extract very long DNA fragments from metagenomic sample; even on the gentlest DNA extractions, the mean fragment length (*μ*_*FL*_) is usually at most 10 to 20 kb. Our simulated data of *μ*_*FL*_ from 5 to 100 kb showed that the assembly was not sensitive to *μ*_*FL*_. In some special cases, extremely long DNA fragments could improve the assemblies of *M*_*sim*_ microbes with high repeat rates. For example, ${\mu}_{FL}^{100}$ (*μ*_*FL*_ = 100 kb) improved the contigs NG50 (3.17 Mb, Fig. 2k) and NGA50 (2.71 Mb, Fig. 2l) of NC_018014, which was the one with the highest repeat rate (18.3%).

### Barcode specificity is important in microbial deconvolution

For human genome sequencing, each partition contains ten fragments (*N*_*F/P*_ = 10) on average [14]. *N*_*F/P*_ is supposed to be larger (*N*_*F/P*_ = 40) for metagenomic sequencing due to the limited fragment size (W*μ*_*FL*_ = 11.15 kb) and relatively small microbial genome size (Table S5). Large *N*_*F/P*_ also increases the difficulties in recognizing the fragments that short-reads belong to. The assembly on *N*_*F/P1*_, the smallest *N*_*F/P*_ (*N*_*F/P*_ = 10) in simulation, had much better NG50 and NGA50 for most of the *H*_*sim*_ and *M*_*sim*_ microbes (14 out of 18, the remaining 4 microbes are comparable, Fig. 2 o and p). Small *N*_*F/P*_ also failed to assemble *L*_*sim*_ microbes (Fig. 2 m and n).

### Assembly on Illumina short-reads and PacBio CCS long-reads

Illumina short-read sequencing is a mainstream technology for metagenomic sequencing, but its quality for metagenome assembly is unsatisfactory due to the lack of long-range connectivity. We downloaded the short-read data of the mock community from the Sequence Read Archive [15] (the “Methods” section) and performed an assembly. The assembly on linked-reads (total aligned length 52.04 Mb; genomic coverage 77.20%) is much better than that on short-reads (total aligned length 38.13 Mb; genomic coverage 56.69%, NG50 and NGA50, see Figure S3). For human gut microbiome, the assembly from 8.8 Gb short-reads showed a comparable number of bins (53) and total assembly length (145.50 Mb vs. 159.40 Mb for S*C*_*R4*_, Table 1). However, the short-read assembly generated no bins with high-quality because it had known issue to detect rRNAs and tRNAs [16, 17] (Table 1). S*C*_*F8*_, with the worst N50 in linked-read assembly, was also 4.49 times (115.29 kb vs. 25.69 kb) greater than Illumina short-reads. The average bin contamination rate of 17.39% for the assembly from short-reads was also much worse than linked-reads (Table S6).

In mock community, we further compared linked-reads to PacBio CCS, which have both extreme long (N50 = 9.08Kb) and highly accurate (> 99% base accuracy) reads. The total aligned length and genomic coverage were comparable between CCS reads (54.04 Mb and 78.68%) and MS*C*_*1*_ (52.02 Mb and 77.18%), but CCS reads improved the contig continuity substantially (Figure S4).

### Comparison to human genome parameter statistics

10x linked-read sequencing was originally developed for human genome assembly, so we compared the parameter distributions between human genome and human gut microbiome. Because no reference genome was available for human gut microbiome, we collected the sequences of all the non-redundant high-quality bins from *SC*_*F-*_ and *SC*_*R-*_ datasets as “pseudo” reference genomes and reconstructed 15,994,284 long fragments (> 2 kb). For human gut microbiome, *C*_*R*_ was comparable (*C*_*R*_ 0.30X vs. 0.32X, Table S5), and *C*_*F*_ was 6.26 times larger than human genome (NA24385, *C*_*F*_ 595.85X vs. 95.20X, Table S5); also, the DNA fragments were obviously much shorter (*μ*_*FL*_ 7.91 kb vs. 28.06 kb; W*μ*_*FL*_ 11.15 kb vs. 44.53 kb, Table S5, Figure S5 and S6).

## Discussion

Human microbiota provide rich information to understand microbial activities impacting human health and disease. Projects such as HMP (Human Microbiome Project) [12] and MetaHIT (Metagenomics of the Human Intestinal Tract) [18] have been proposed to collect microbiomes from diverse places of human body and aimed to understand their compositions and functions. De novo metagenome assembly on short-reads is commonly used to assemble microbial genomes from a mixture of culture-free microbes. Although it has been widely applied to assemble thousands of bacterial genomes [19, 20], there are four difficulties that remain: (1) assembly for low-abundance microbes; (2) repetitive sequences assembly such as 16S, 23S rRNA; (3) assembly of regions with genetic variation; (4) strain level assembly based on haplotype phasing. Besides metaSPAdes used in the current study, IDBA-UD [2] and MEGAHIT [3] were also tested and achieved comparable results with metaSPAdes. They all showed much worse assembly than linked-reads (Table S6).

Long-read sequencing has the potential to assemble more complete genomes and is believed to dominate the field in the future. However, linked-reads are still worth to be considered as a transitional technology. First, both PacBio and Oxford Nanopore are several times more costly than 10x linked-reads (especially for library preparation). Second, high base error rate of long-reads lacks strength for haplotype phasing and strain level assembly. Third, clinical samples benefit from the small amount of input DNA required by linked-read sequencing. A previous study also observed some high-quality bins generated by linked-reads missed in long-reads assembly [7].

In this study, we comprehensively investigated the four parameters of linked-read sequencing on metagenome assembly, which could be fine-tuned in either library preparation or short-read sequencing. Read depth *C* and microbial abundance are the two most important parameters to determine genome coverage and the number of bins annotated as draft genomes. Low-abundance microbes were almost impossible to be assembled by any of the technologies; the assemblies of medium-abundance microbes were substantially improved by deep *C*, and they were fairly stable for high-abundance ones.

According to our observation, *C* should be chosen from 120X to 400X to optimize the assembly quality. There is a tradeoff between *C*_*F*_ and *C*_*R*_, where deep *C*_*F*_ can generate more high-quality bins and *C*_*R*_ controls total assembly length. Large *μ*_*FL*_ enables DNA fragments spanning distant contigs, but it is unnecessary to produce extremely long fragments for microbial genomes. The repetitive sequences spread in microbial genomes are usually short (e.g.,16S: ~ 1.5 kb, 23S: ~ 2.9 kb), which could be resolved by assembling the co-barcoded reads with small *N*_*F/P*_.

Athena-meta includes four steps: (1) generate “seed” contigs using short-reads without barcodes; (2) link contigs into scaffold graph using aligned paired-end reads; (3) local assembly by recruiting co-barcoded reads that spanning both “seed” contigs; (4) pool and assemble the locally assembled sequences and “seed” contigs. We can link and interpret our observations with the corresponding strategies in Athena-meta. *C* is critical to construct “seed” contigs, as high-quality seed contigs are the prerequisite for local assembly using co-barcoded reads. *C*_*R*_ and *C*_*F*_ impact reconstruction of long DNA fragments, and the probability of two distant contigs spanned by the same fragment, respectively. Small *N*_*F/P*_ can reduce off-target reads and make local-assembly more efficiently. Our study revealed that the probable best practice in using linked-reads for metagenome assembly is to merge the linked-reads from multiple libraries, where each has sufficient *C*_*R*_ but a smaller amount of input DNA.

## Methods

### Simulate linked-reads for microbes with uneven abundance

LRTK-SIM [11] was initially built for human diploid assembly by simulating 10x linked-reads. In this study, we extended it to allow genomes with uneven depth to reflect different microbial abundance (Figure S7). We downloaded the reference genomes (denoted as *M*) of 23 bacterial and 3 archaeal strains from MBARC-26 [21] and categorized them into *L*_*sim*_ (*Molarity* < 10^−15^), *M*_*sim*_ (10^−15^ < *Molarity* < 10^−14^) and *H*_*sim*_ (*Molarity* > 10^−14^) (Table S1). The molarity was normalized to sum to 1 as microbial relative abundance (${\sum}_{i=1}^{26}{A}_i=1$), and *C*_*F*_ for microbe *i* (*C*_*Fi*_) was calculated as *C*_*Fi*_ = *C*_*F*_ × *A*_*i*_ × 26 (*C*_*F*_ was predefined). The total fragment length for microbe *i* (*M*_*i*_) was *C*_*Fi*_× *L*_*i*_, where *L*_*i*_ was genome size of *M*_*i*_. The estimated input nucleotides were calculated as ${\sum}_{i=1}^M{A}_i\times {L}_i\times 26$. We simulated a wide range of *C*_*F*_ (from 28X to 333X), *C*_*R*_ (from 0.064X to 0.77X), *μ*_*FL*_ (from 5 to 100 kb), and *N*_*F/P*_ (from 10 to 160) to investigate their impact on metagenome assembly (Table S1).

### DNA extraction, library preparation, and sequencing

For mock community, DNA from ATCC 20 strain staggered mix genomic material (ATCC-MSA1003) was extracted without size-selection. For human gut microbiome from stool sample, we extracted the DNA using Qiagen QiAaMP Stool Mini Kit and removed the DNA fragments below 5 kb. After that, the molecular weight of isolated DNA was assayed by pulsed-field electrophoresis. For 10x Chromium library preparation, 1 ng of isolated high molecular weight DNA was denatured according to the manufacturer recommendations, added to the reaction master mix and mixed with gel bead and emulsification oil to generate droplets within a Chromium Genome chip. The rest part of library preparation was done following the manufacturer protocol (Chromium Genome v1, PN-120229).

The two libraries were sequenced by Illumina XTen with 2 × 150 bps paired-end reads, respectively. The DNA of human gut microbiome was also prepared for standard Illumina XTen short-read sequencing.

### DNA long fragment reconstruction and linked-read subsampling

Long Ranger v2.2.1 [22] was used to correct barcode base errors, calculate PCR duplication rate, and perform barcode-aware linked-read alignment. BWA-MEM v0.7.17 [23] was adopted to align short-reads and linked-reads without barcodes. Long DNA fragments were reconstructed according to the mapping coordinates of co-barcoded short-reads. The linked-reads were sorted by barcode first and then by their mapping coordinates. Long DNA fragments were reconstructed by greedy extension and terminated if the nearest co-barcoded read was > 50 kb away. Each fragment must include at least two co-barcoded read pairs and have a minimum length of 2 kb.

### Metagenome assembly

For linked-read assembly, the linked-reads without barcodes were first assembled into seed contigs by metaSPAdes v3.11.1 [4] with default parameters and aligned to contigs by BWA-MEM v0.7.17. Athena-meta v1.3 was applied for local assembly by collecting co-barcoded reads shared by two “seed” contigs in scaffold graph (Figure S8). For mock community, the Illumina short-reads (SRR8359173) and PacBio CCS reads (SRR9202034 and SRR9328980) were assembled by metaSPAdes v3.12.0 and Canu v2.0 [24], respectively. The command lines were included in the Supplementary Note.

### Assembly evaluation

We implemented a pipeline (Figure S9) to compare different metagenome assemblies by integrating off-the-shelf software and in-house scripts. First, MaxBin v2.2.4 [25] grouped contigs (longer than 1 kb) into bins, and their completeness and contaminations were assessed by CheckM v1.0.12 [26]. Quast v5.0.0 [27] calculated basic statistics such as contig N50, NG50, NGA50, total aligned length, and genomic coverage; Aragorn v1.2.38 [28] and Barrnap (https://github.com/tseemann/barrnap) were used to infer tRNA and rRNA (5S, 16S, and 23S), respectively; Kraken v0.10.6 [29] annotated taxonomic classification of bins based on its built-in database MiniKrakenDB. The bin abundance was calculated by $\frac{\mathrm{size}\left(\mathrm{bin}\right)\mathrm{dp}\left(\mathrm{bin}\right)}{\mathrm{len}\left(\mathrm{read}\right)\mathrm{sum}\left(\mathrm{read}\right)}$. For each bin, size(bin) is its total nucleotides, dp(bin) denotes its read depth, len(read) is short-read length, and sum(read) is total number of aligned short-reads. Bins were recognized as draft genomes if they were classified as high-quality (completeness > 90%, contamination < 5%, presence of the 5S, 16S, 23S rRNAs, and at least 18 tRNA), medium-quality (completeness ≥ 50% and contamination < 10%), and low-quality (completeness < 50% and contamination < 10%). The command lines were included in the Supplementary Note.

## Conclusion

In this study, we comprehensively investigated four parameters of linked-read sequencing on metagenome assembly and compared with Illumina short-reads and PacBio CCS reads. Our study revealed that the probable best practice in using linked-reads for metagenome assembly is to merge the linked-reads from multiple libraries, where each has sufficient *C*_*R*_ but a smaller amount of input DNA.

## Supplementary information

**Additional file 1: Table S1**. Parameter configurations of the simulated data sets. **Table S2**. Summary of the microbes in MBARC-26. Microbes were classified as High- (*H*_*sim*_, *Molarity* > 10^−14^), Medium- (*M*_*sim*_,10^−15^ < *Molarity* < 10^−14^) and Low- (*L*_sim_, *Molarity* < 10^−15^) abundance based on their molarities. **Table S3**. Summary of 20 microbes in ATCC MSA-1003. Microbes were classified as UltraHigh- (*UH*_*mock*_, percentage = 18%), High- (*H*_*mock*_, percentage = 1.8%), Medium- (*M*_*mock*_, percentage = 0.18%) and Low- (*L*_*mock*_, percentage = 0.02%) abundance according to their mixture amount. **Table S5**. The key parameters of 10x linked-read sequencing for human gut metagenome and human genome. **Table S6**. The performance of metaSPAdes, MEGAHIT and IDBA-UD on short-read sequencing from human gut microbiome.

**Additional file 2: Table S4**. Annotations of assemblies for the subsampled linked-reads from human gut microbiome.

**Additional file 3: Table S7** A summary of 65,535 microbes in human microbiome project covered by 10x linked-reads from human gut microbiome. **Table S8**. A summary of 1,285 microbes in human microbiome project covered by 10x linked-reads of human gut microbiome with genomic coverage>90% and sequencing depth > 20X.

**Additional file 4: Figure S1**. Distributions of bin completeness and contamination of S*C*_F-_ and S*C*_R-_ of human gut microbiome data. **Figure S2**. Upset plots for the shared genus (A: *SC*_*F-*_, C: *SC*_*R-*_) and species (B: *SC*_*F-*_, D: *SC*_*R-*_) of different subsampling datasets. **Figure S3**. Comparison of the contig NG50 and NGA50 between Illumina short-reads (Illumina) and 10x linked-reads (MS*C*_*1*_) from the mock community. **Figure S4**. Comparison of the contig NG50 and NGA50 between PacBio CCS reads (CCS) and 10x linked-reads (MS*C*_*1*_) from the mock community. **Figure S5**. Parameter distributions of linked-read sequencing from human gut microbiome. PDF: probability density function; CDF: cumulative density function. **Figure S6**. Parameter distributions of linked-read sequencing from human genome (NA24385). PDF: probability density function; CDF: cumulative density function. **Figure S7**. Workflow of LRTK-SIM to simulate linked-reads for microbial genomes with uneven depth. **Figure S8**. Workflow of linked-reads metagenome assembly on simulated 10x linked-reads. **Figure S9**. Workflow for evaluating and comparing different metagenome assemblies. **Figure S10**. The distributions of genomic coverage and read depth for the microbes in human microbiome project according to the alignment of the linked-reads from human gut microbiome. CDF: cumulative density function. Supplementary Note: 1. Complexity and statistics for linked-reads from human gut microbiome. 2. Command lines adopted for the analysis.

## Data Availability

The source codes of LRTK-SIM and analysis pipeline are publicly available at https://github.com/zhanglu295/LRTK-SIM and https://github.com/liaoherui/MetaComp, respectively. The raw sequencing data are deposited in the Sequence Read Archive and the corresponding BioProject accession number is PRJNA573416 and PRJNA647353. For mock community, the Illumina short-reads and PacBio CCS reads are available in Sequence Read Archive; the accession numbers are SRR8359173, SRR9202034, and SRR9328980. All the command lines were included in the Supplementary Note.

## Abbreviations

*C*_*R*_: Average depth of short reads per fragment
*C*_*F*_: Average physical depth of the genome by long DNA fragments
*N*_*F/P*_: Number of fragments per partition
*μ*_*FL*_: Average unweighted DNA fragment length
W*μ*_*FL*_: Length-weighted average of DNA fragment length
*C*: Total sequencing depth, *C*_*R*_ x *C*_*F*_ = *C*
MS*C*_*R-*_ and MS*C*_*F-*_: Subsampling short-reads and reconstructed long DNA fragment from mock community linked-read data (“-” represents the reciprocal of sequenced lanes)
S*C*_*R-*_ and S*C*_*F-*_: Subsampling short-reads and barcodes from human gut microbiome linked-read data (“-” represents the reciprocal of sequenced lanes)
*L*_*sim*_/*M*_*sim*_/*H*_*sim*_: Low-/medium-/high-abundance microbes in the simulated data
*L*_*mock*_/*M*_*mock*_/*H*_*mock*_/*UH*_*mock*_: Low-/medium-/high-/ultrahigh-abundance microbes in the mock community
