# Supplementary material for: A comprehensive investigation of metagenome assembly by linked-read sequencing
Source: Microbiome. 2020 Nov 11;8:156. doi: 10.1186/s40168-020-00929-3 (PMC7659138; doi:10.1186/s40168-020-00929-3)
Supplement: Supplementary file 2 — Additional file 1: Table S1. Parameter configurations of the simulated data sets. Table S2. Summary of the microbes in MBARC-26. Microbes were classified as High- (Hsim, Molarity > 10−14), Medium- (Msim,10−15 < Molarity < 10−14) and Low- (Lsim, Molarity < 10−15) abundance based on their molarities. Table S3. Summary of 20 microbes in ATCC MSA-1003. Microbes were classified as UltraHigh- (UHmock, percentage = 18%), High- (Hmock, percentage = 1.8%), Medium- (Mmock, percentage = 0.18%) and Low- (Lmock, percentage = 0.02%) abundance according to their mixture amount. Table S5. The key parameters of 10x linked-read sequencing for human gut metagenome and human genome. Table S6. The performance of metaSPAdes, MEGAHIT and IDBA-UD on short-read sequencing from human gut microbiome. [file 40168_2020_929_MOESM1_ESM.docx]

**Supplementary Table**

| **Parameters** | **ID** | ***N_F/P_*** | $\boldsymbol{\mu}_{\boldsymbol{FL}}$  **(kb)** | ***C_F_* (X)** | ***C_R_***  **(X)** | ***C***  **(*C_F_* × *C_R_*)** |
| --- | --- | --- | --- | --- | --- | --- |
| *C_F_* | $C_{F}^{28}$ | 50 | 10 | 28 | 0.36 | 10 |
|  | $C_{F}^{83}$ | 50 | 10 | 83 | 0.36 | 30 |
|  | $C_{F}^{156}$ | 50 | 10 | 156 | 0.36 | 56 |
|  | $C_{F}^{217}$ | 50 | 10 | 217 | 0.36 | 78 |
|  | $C_{F}^{333}$ | 50 | 10 | 333 | 0.36 | 120 |
| *C_R_* | $C_{R}^{0.064}$ | 50 | 10 | 156 | 0.064 | 10 |
|  | $C_{R}^{0.19}$ | 50 | 10 | 156 | 0.19 | 30 |
|  | $C_{R}^{0.36}$ | 50 | 10 | 156 | 0.36 | 56 |
|  | $C_{R}^{0.5}$ | 50 | 10 | 156 | 0.5 | 78 |
|  | $C_{R}^{0.77}$ | 50 | 10 | 156 | 0.77 | 120 |
| $\mu_{FL}$ | $\mu_{FL}^{5}$ | 100 | 5 | 156 | 0.36 | 56 |
|  | $\mu_{FL}^{10}$ | 50 | 10 | 156 | 0.36 | 56 |
|  | $\mu_{FL}^{20}$ | 25 | 20 | 156 | 0.36 | 56 |
|  | $\mu_{FL}^{50}$ | 10 | 50 | 156 | 0.36 | 56 |
|  | $\mu_{FL}^{100}$ | 5 | 100 | 156 | 0.36 | 56 |
| *N_F/P_* | $N_{F/P}^{10}$ | 10 | 10 | 156 | 0.36 | 56 |
|  | $N_{F/P}^{40}$ | 40 | 10 | 156 | 0.36 | 56 |
|  | $N_{F/P}^{80}$ | 80 | 10 | 156 | 0.36 | 56 |
|  | $N_{F/P}^{120}$ | 120 | 10 | 156 | 0.36 | 56 |
|  | $N_{F/P}^{160}$ | 160 | 10 | 156 | 0.36 | 56 |

**Table S1.** Parameter configurations of the simulated data sets.

**Table S2. Summary of the microbes in MBARC-26.** Microbes were classified as High- (*H_sim_*, $Molarity>{10}^{-14}$), Medium- (*M_sim_*,${10}^{-15}<Molarity<{10}^{-14}$) and Low- (*L*_sim_, $Molarity<{10}^{-15}$) abundance based on their molarities.

| **Organism** | **GenBank Accession** | **Genome Size (bp)** | **Molarity** | **Classification** |
| --- | --- | --- | --- | --- |
| Terriglobus roseus DSM 18391 (AD) | NC_018014 | 5227858 | 4.79E-15 | *M_sim_* |
| Corynebacterium glutamicum ATCC 13032 (AT) | NC_003450 | 3309401 | 4.91E-16 | *L_sim_* |
| Nocardiopsis dassonvillei DSM 43111 (AT) | NC_014211 | 6543312 | 2.67E-17 | *L_sim_* |
| Olsenella uli DSM 7084 (AT) | NC_014363 | 2051896 | 3.40E-14 | *H_sim_* |
| Segniliparus rotundus DSM 44985 (AT) | NC_014168 | 3157527 | 1.22E-14 | *H_sim_* |
| Echinicola vietnamensis DSM 17526 (B) | NC_019904 | 5608040 | 1.26E-15 | *M_sim_* |
| Meiothermus Silvanus DSM 9946 (D) | NC_014212 | 3721669 | 4.38E-14 | *H_sim_* |
| Clostridium perfringens ATCC 13124 (F) | NC_008261 | 3256683 | 5.20E-16 | *L_sim_* |
| Clostridium thermocellum ATCC 27405 (F) | NC_009012 | 3843301 | 4.40E-16 | *L_sim_* |
| Desulfosporosinus acidiphilus SJ4 DSM 22704 (F) | NC_018068 | 4991181 | 2.68E-14 | *H_sim_* |
| Desulfosporosinus meridiei DSM 13257 (F) | NC_018515 | 4873567 | 9.89E-15 | *M_sim_* |
| Desulfotomaculum gibsoniae DSM 7213 (F) | NC_021184 | 4855529 | 2.93E-14 | *H_sim_* |
| Streptococcus pyogenes M1 GAS SF370 (F) | NC_002737 | 1852441 | 1.53E-15 | *M_sim_* |
| Thermobacillus composti KWC4, DSM 18247 (F) | NC_019897 | 4355525 | 2.39E-16 | *L_sim_* |
| Escherichia coli K-12, MG1655 (P) | NC_000913 | 4639675 | 3.90E-16 | *L_sim_* |
| Frateuria aurantia DSM 6220 (P) | NC_017033 | 3603458 | 2.84E-14 | *H_sim_* |
| Hirschia baltica ATCC 49814 (P) | NC_012982 | 3540114 | 1.78E-14 | *H_sim_* |
| Pseudomonas stutzeri RCH2 (P) | NC_019936 | 4600489 | 1.21E-14 | *H_sim_* |
| Salmonella bongori NCTC 12419 (P) | NC_015761 | 4460105 | 1.72E-16 | *L_sim_* |
| Salmonella enterica subsp. arizonae serovar RSK2980 (P) | NC_010067 | 4600800 | 6.69E-16 | *L_sim_* |
| Spirochaeta smaragdinae DSM 11293 (S) | NC_014364 | 4653970 | 2.78E-14 | *H_sim_* |
| Fervidobacterium pennivorans DSM 9078 (T) | NC_017095 | 2166381 | 6.21E-14 | *H_sim_* |
| Coraliomargarita akajimensis DSM 45221 (V) | NC_014008 | 3750771 | 6.85E-15 | *M_sim_* |
| Halovivax ruber XH-70 (E) | CP003050 | 3223876 | 2.34E-14 | *H_sim_* |
| Natronobacterium gregoryi SP2 (E) | NC_019792.1 | 3788356 | 3.01E-14 | *H_sim_* |
| Natronococcus occultus DSM 3396 (E) | NC_019974.1 | 4314118 | 2.15E-14 | *H_sim_* |

| **Organism** | **ATCC ID** | **Percentage** | **Classification** |
| --- | --- | --- | --- |
| Bacteroides vulgatus | ATCC_8482 | 0.02% | *L_mock_* |
| Bifidobacterium adolescentis | ATCC_15703 | 0.02% | *L_mock_* |
| Deinococcus radiodurans | ATCC_BAA816 | 0.02% | *L_mock_* |
| Enterococcus faecalis | ATCC_47077 | 0.02% | *L_mock_* |
| Schaalia odontolytica | ATCC_17982 | 0.02% | *L_mock_* |
| Acinetobacter baumannii | ATCC_17978 | 0.18% | *M_mock_* |
| Cutibacterium acnes | ATCC_11828 | 0.18% | *M_mock_* |
| Helicobacter pylori | ATCC_700392 | 0.18% | *M_mock_* |
| Lactobacillus gasseri | ATCC_33323 | 0.18% | *M_mock_* |
| Neisseria meningitidis | ATCC_BAA335 | 0.18% | *M_mock_* |
| Bacillus cereus | ATCC_10987 | 1.8% | *H_mock_* |
| Clostridium beijerinckii | ATCC_35702 | 1.8% | *H_mock_* |
| Pseudomonas aeruginosa | ATCC_9027 | 1.8% | *H_mock_* |
| Staphylococcus aureus | ATCC_BAA1556 | 1.8% | *H_mock_* |
| Streptococcus agalactiae | ATCC_BAA611 | 1.8% | *H_mock_* |
| Escherichia coli | ATCC_700926 | 18% | *UH_mock_* |
| Porphyromonas gingivalis | ATCC_33277 | 18% | *UH_mock_* |
| Rhodobacter sphaeroides | ATCC_17029 | 18% | *UH_mock_* |
| Staphylococcus epidermidis | ATCC_12228 | 18% | *UH_mock_* |
| Streptococcus mutans | ATCC_700610 | 18% | *UH_mock_* |

**Table S3. Summary of 20 microbes in ATCC MSA-1003.** Microbes were classified as UltraHigh- (*UH_mock_*, percentage=18%), High- (*H_mock_*, percentage=1.8%), Medium- (*M_mock_*, percentage=0.18%) and Low- (*L_mock_*, percentage=0.02%) abundance according to their mixture amount.

|  | ***C_F_* (X)** | ***C_R_* (X)** | $\boldsymbol{\mu}_{\boldsymbol{FL}}$ **(kb)** | $\boldsymbol{W\mu}_{\boldsymbol{FL}}$ **(kb)** |
| --- | --- | --- | --- | --- |
| **Metagenome** | 595.85 | 0.30 | 7.91 | 11.15 |
| **Human Genome** | 95.20 | 0.32 | 28.06 | 44.53 |

**Table S5.** The key parameters of 10x linked-read sequencing for human gut metagenome and human genome.

| **Software** | **# of bins** | **Total Length (Mb)** | **High (%)** | **Medium (%)** | **Low (%)** | **Others (%)** |
| --- | --- | --- | --- | --- | --- | --- |
| **metaSPAdes** | 53 | 145.51 | 0 (0) | 16 (30.19) | 16 (30.19) | 21 (39.62) |
| **MEGAHIT** | 53 | 149.96 | 1 (1.89) | 16 (30.19) | 12 (22.64) | 25 (9.43) |
| **IDBA-UD** | 50 | 145.05 | 1 (2) | 13 (26) | 18 (36) | 19 (38) |

**A.** Basic statistics

| **Software** | **Completeness** | **Contamination** | **N50 (High)** | **N50 (Medium)** | **N50 (Low)** |
| --- | --- | --- | --- | --- | --- |
| **metaSPAdes** | 65.71% | 17.39% | 0 | 51.49Kb | 12.43Kb |
| **MEGAHIT** | 71.10% | 17.95% | 4.07Mb | 49.16Kb | 19.35Kb |
| **IDBA-UD** | 69.00% | 17.00% | 4.09Mb | 30.14Kb | 13.51Kb |

**B.** Bin quality

**Table S6.** The performance of metaSPAdes, MEGAHIT and IDBA-UD on short-read sequencing from human gut microbiome.
