# Supplementary material for: A comprehensive investigation of metagenome assembly by linked-read sequencing
Source: Microbiome. 2020 Nov 11;8:156. doi: 10.1186/s40168-020-00929-3 (PMC7659138; doi:10.1186/s40168-020-00929-3)
Supplement: Supplementary file 5 — Additional file 4: Figure S1. Distributions of bin completeness and contamination of SCF- and SCR- of human gut microbiome data. Figure S2. Upset plots for the shared genus (A: SCF-, C: SCR-) and species (B: SCF-, D: SCR-) of different subsampling datasets. Figure S3. Comparison of the contig NG50 and NGA50 between Illumina short-reads (Illumina) and 10x linked-reads (MSC1) from the mock community. Figure S4. Comparison of the contig NG50 and NGA50 between PacBio CCS reads (CCS) and 10x linked-reads (MSC1) from the mock community. Figure S5. Parameter distributions of linked-read sequencing from human gut microbiome. PDF: probability density function; CDF: cumulative density function. Figure S6. Parameter distributions of linked-read sequencing from human genome (NA24385). PDF: probability density function; CDF: cumulative density function. Figure S7. Workflow of LRTK-SIM to simulate linked-reads for microbial genomes with uneven depth. Figure S8. Workflow of linked-reads metagenome assembly on simulated 10x linked-reads. Figure S9. Workflow for evaluating and comparing different metagenome assemblies. Figure S10. The distributions of genomic coverage and read depth for the microbes in human microbiome project according to the alignment of the linked-reads from human gut microbiome. CDF: cumulative density function. Supplementary Note: 1. Complexity and statistics for linked-reads from human gut microbiome. 2. Command lines adopted for the analysis. [file 40168_2020_929_MOESM4_ESM.docx]

**
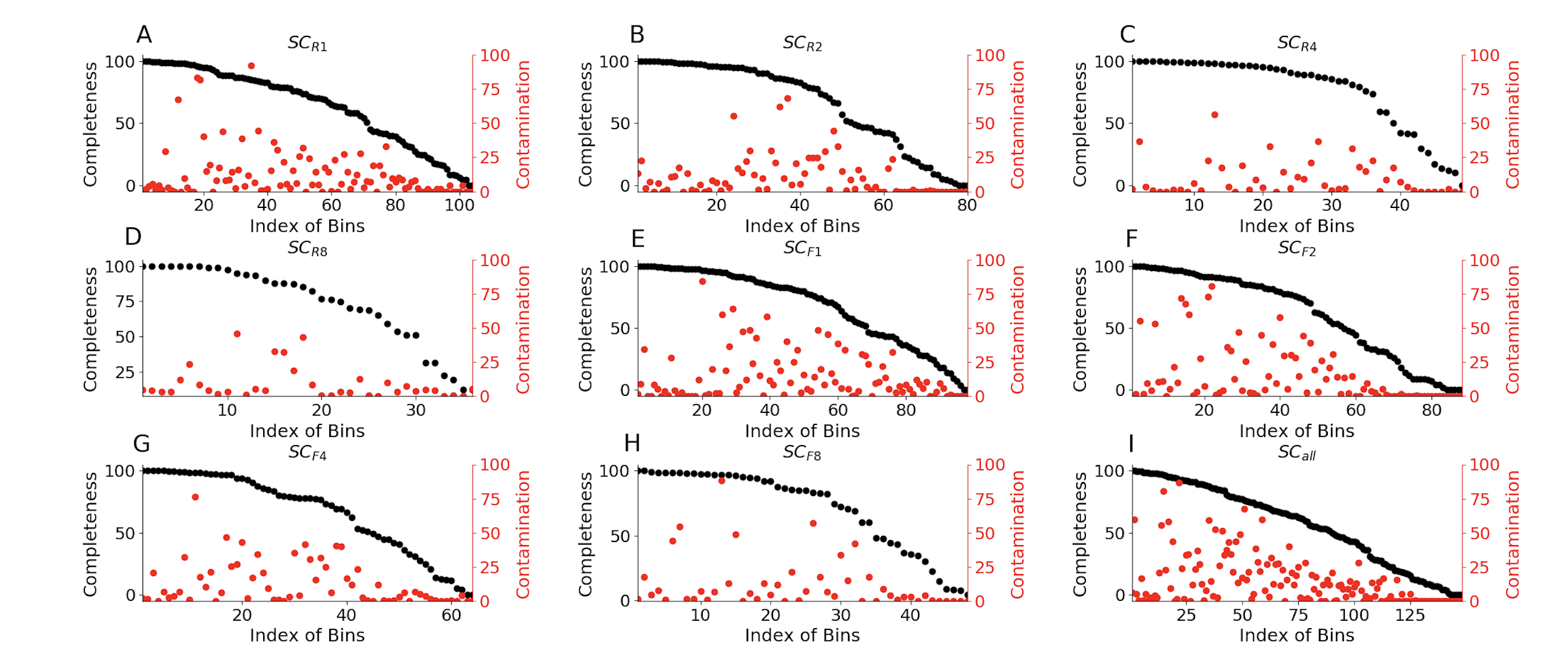
**

**Figure S1.** Distributions of bin completeness and contamination of S*C*_F-_ and S*C*_R-_ of human gut microbiome data.


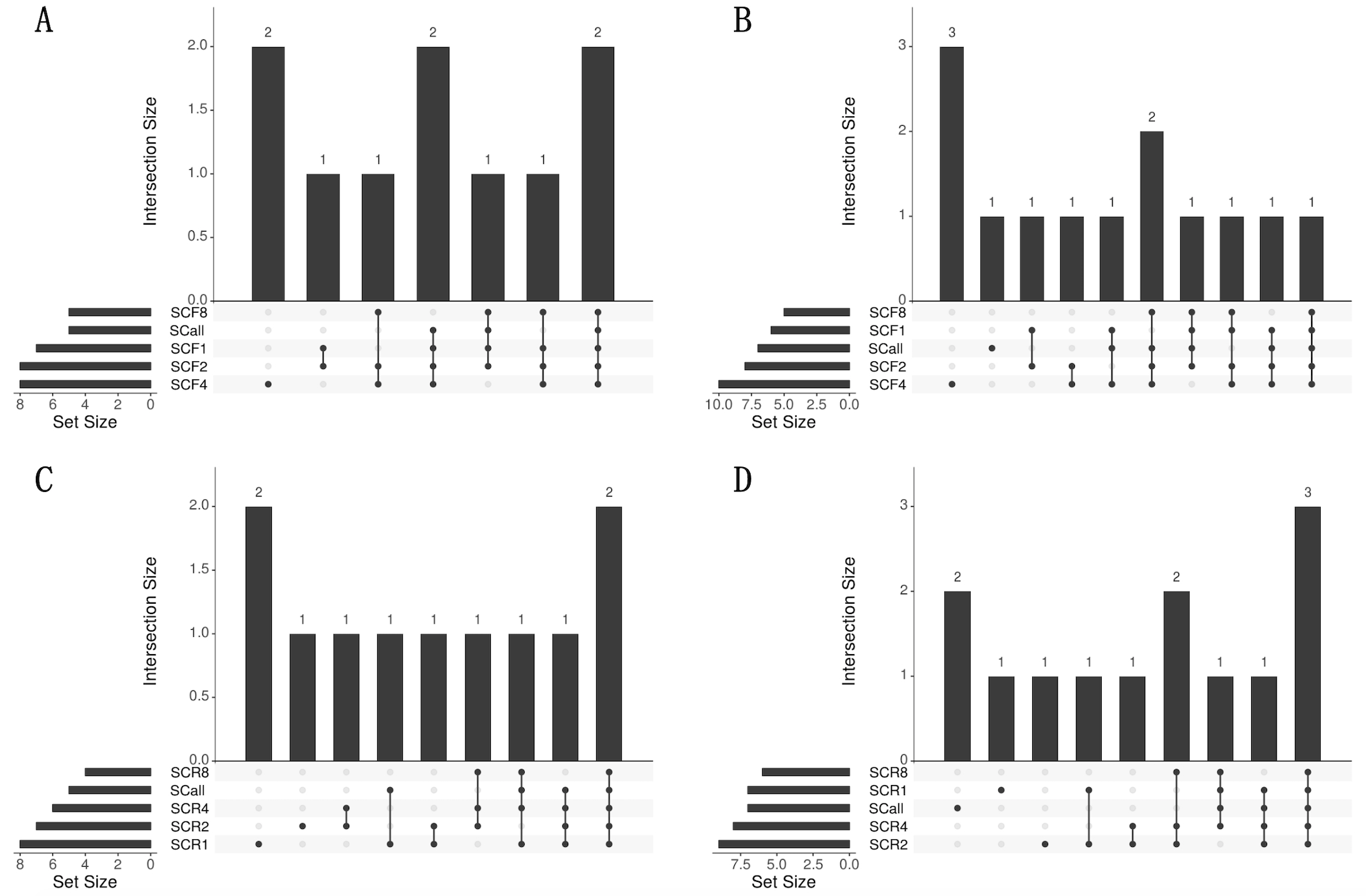


**Figure S2.** Upset plots for the shared genus (A: S*C_F-_*, C: S*C_R-_*) and species (B: S*C_F-_*, D: S*C_R-_*) of different subsampling datasets.


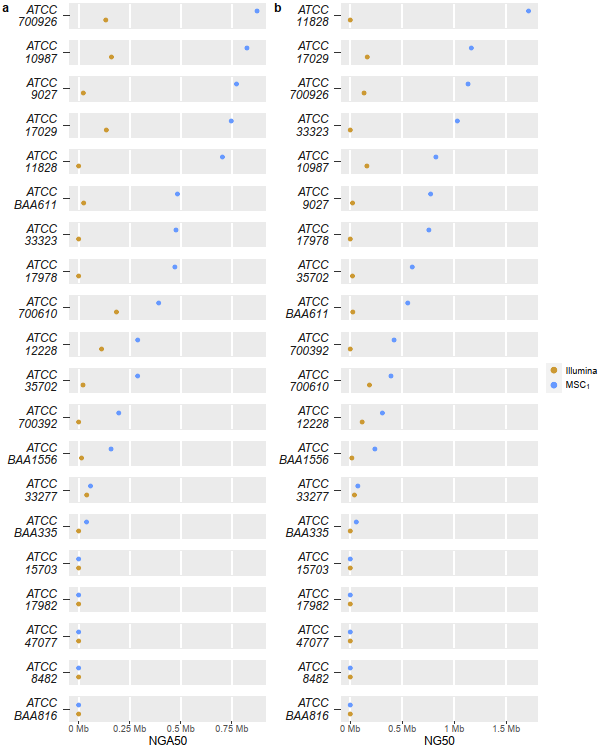


**Figure S3.** Comparison of the contig NG50 and NGA50 between Illumina short-reads (Illumina) and 10x linked-reads (MS*C_1_*) from the mock community.


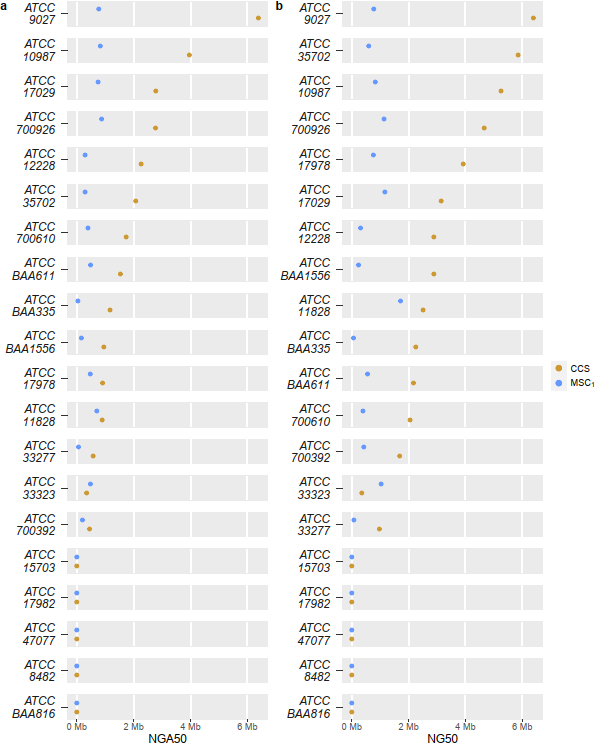


**Figure S4.** Comparison of the contig NG50 and NGA50 between PacBio CCS reads (CCS) and 10x linked-reads (MS*C_1_*) from the mock community.

**Figure S5.** Parameter distributions of linked-read sequencing from human gut microbiome. PDF: probability density function; CDF: cumulative density function.

**Figure S6.** Parameter distributions of linked-read sequencing from human genome (NA24385). PDF: probability density function; CDF: cumulative density function.

**
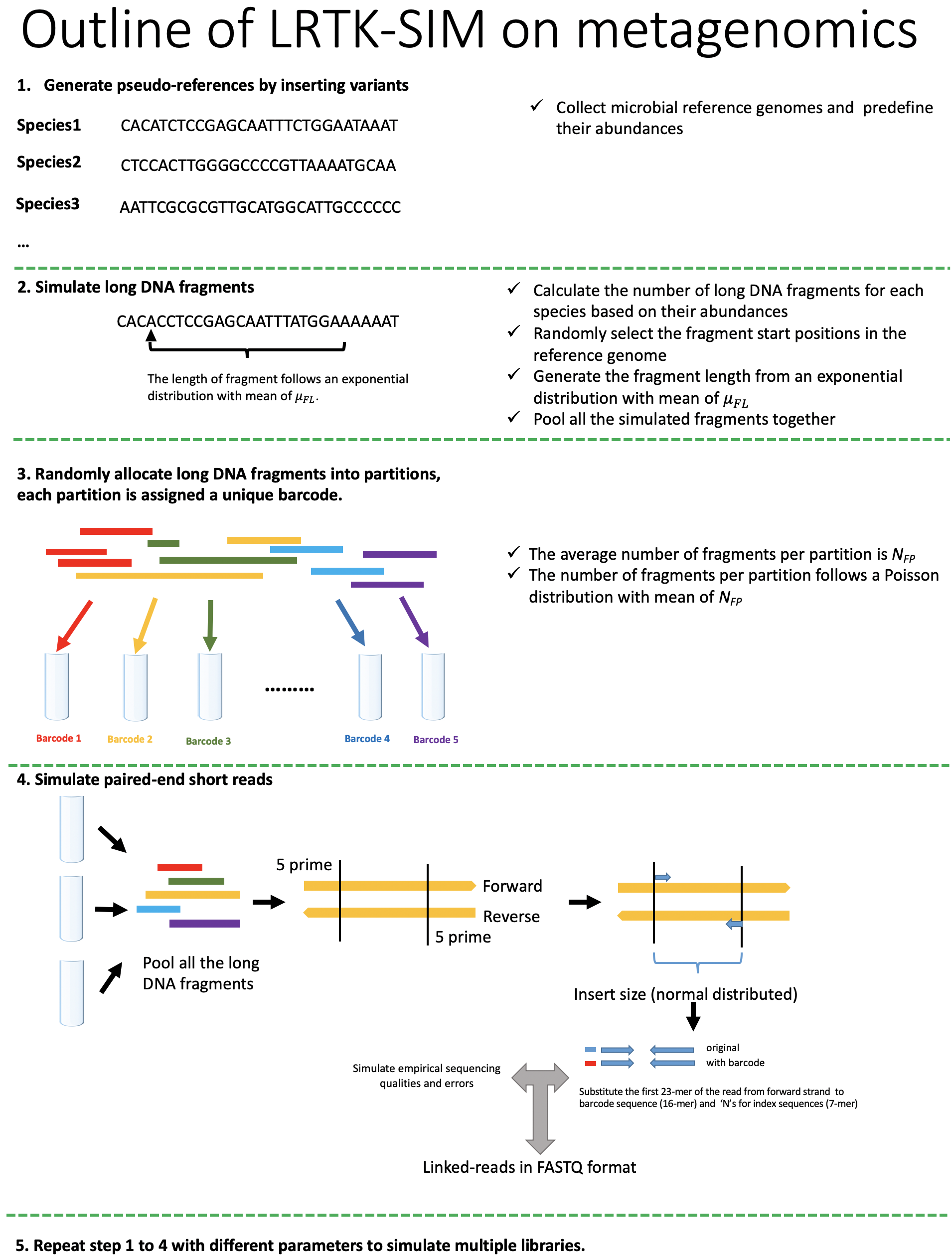
**

**Figure S7.** Workflow of LRTK-SIM to simulate linked-reads for microbial genomes with uneven depth.

**
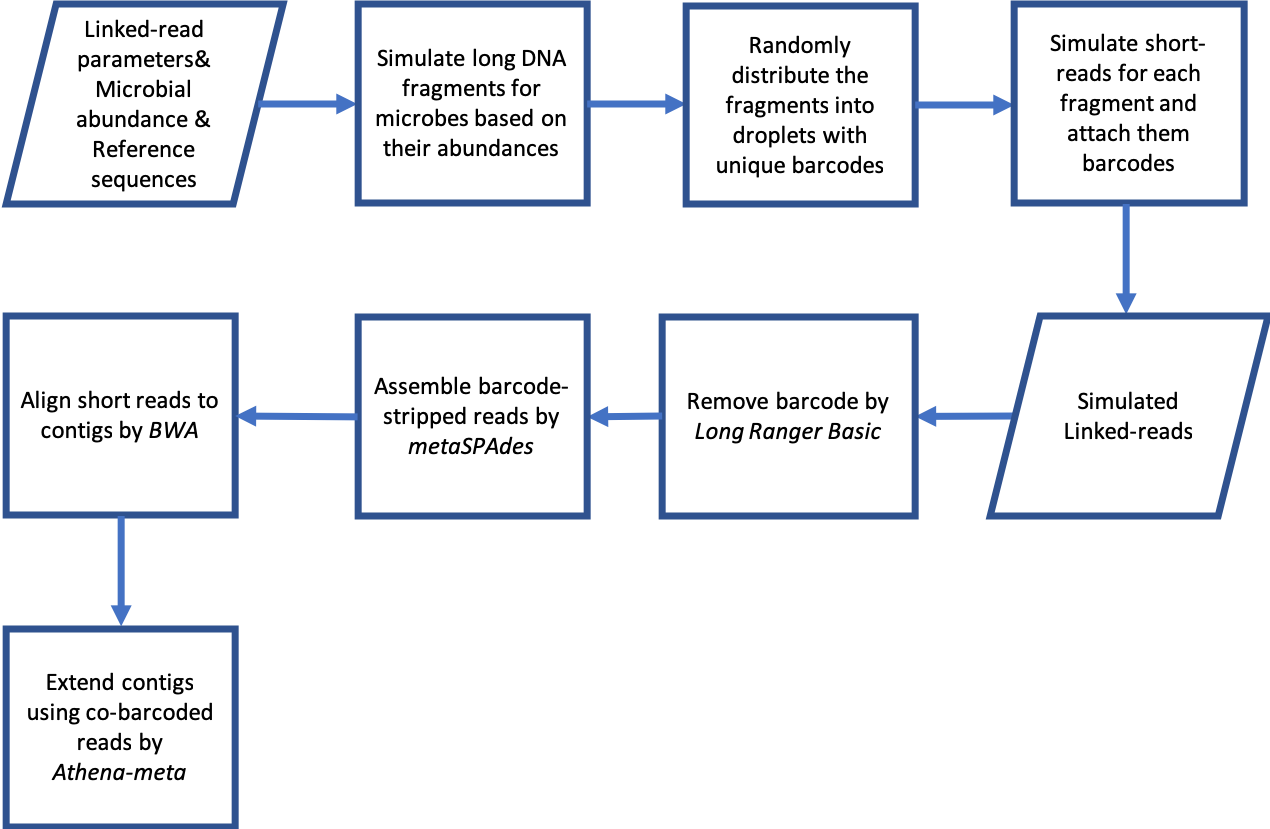
**

**Figure S8.** Workflow of linked-reads metagenome assembly on simulated data.


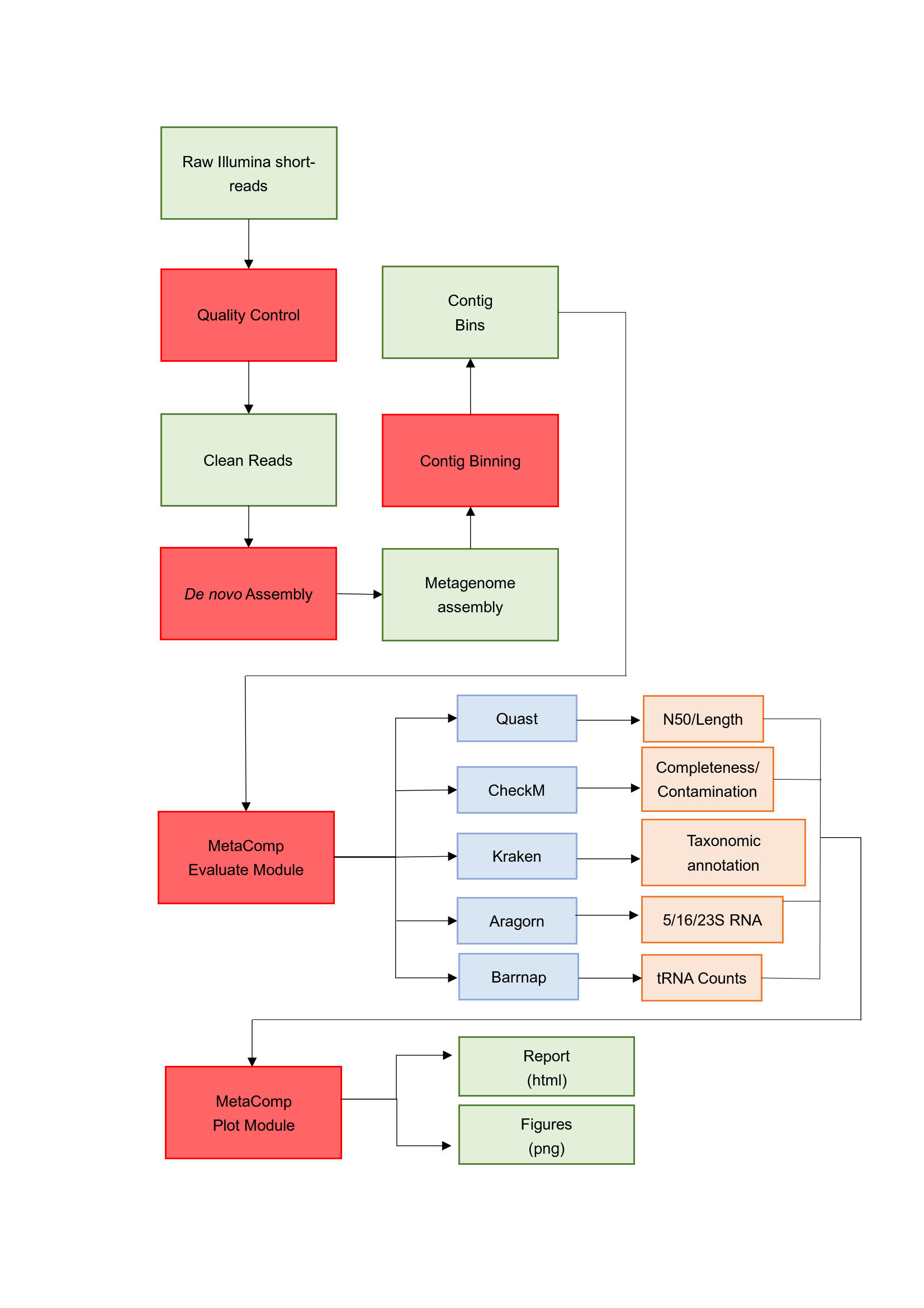


**Figure S9.** Workflow for evaluating and comparing different metagenome assemblies.


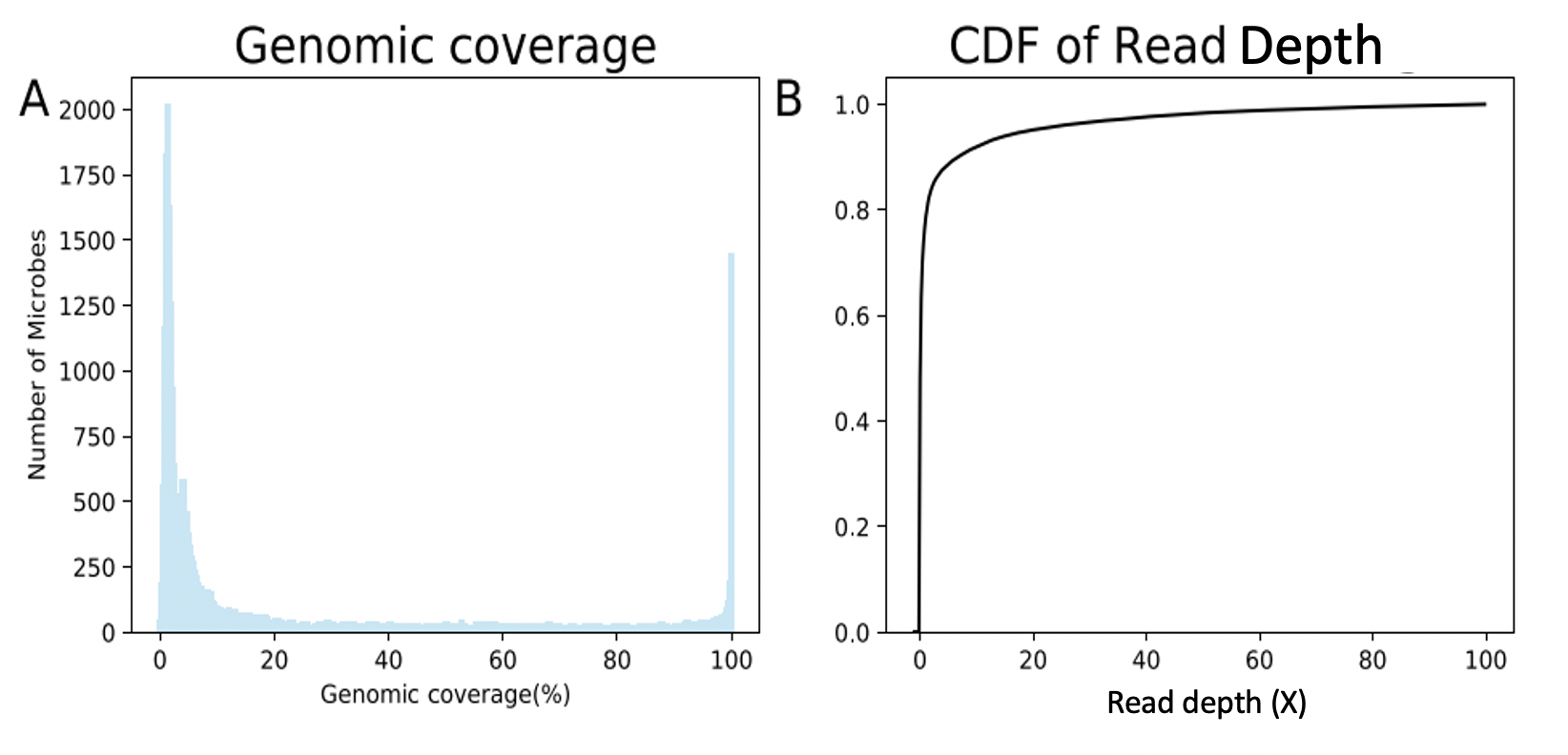


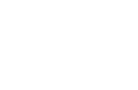


Depth


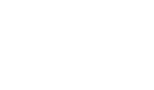


Read depth (X)

**Figure S10.** The distributions of genomic coverage and read depth for the microbes in human microbiome project according to the alignment of the linked-reads from human gut microbiome. CDF: cumulative density function.

**Supplementary Note**

**1. Complexity and statistics for linked-reads from human gut microbiome**

The barcode sequences of 4.7% linked-reads were discarded due to sequencing errors. These barcode-stripped reads could be aligned to 65,535 microbial genomes (34.85%; **Table S7** and **Figure S10**) from human microbiome project and 1,285 of them were well covered (genomic coverage>90%, read depth>20X, **Table S8**), suggesting a high diversity of metagenomic composition between individuals. The PCR duplication rate was 24.89%.

**2. Command lines adopted for the analysis**

A. Correct barcode base error and generate barcode-stripped reads

longranger basic --id=mock --fastqs=./input_fastq

B. Barcode-aware linked-reads alignment and calculate PCR duplication rate

longranger align --id=mock_alignment --reference=refdata-atcc20-refs --fastqs=./input_fastq

C. Assemble barcode-stripped reads

metaspades.py --12 /path/to/reads -o /path/to/metaspades/out

D. Align barcode-stripped reads to contigs

bwa index /path/to/metaspades/out/contigs.fasta

bwa mem -C -p /path/to/metaspades/out/contigs.fasta /path/to/reads | samtools sort -o align-reads.metaspades-contigs.bam –

samtools index align-reads.metaspades-contigs.bam

E. Assemble linked-reads by Athena-meta

athena-meta --config /path/to/config.json

F. Assemble PacBio ccs reads by Canu

canu -p atcc20 -d canu_grid genomeSize=66930628 correctedErrorRate=0.025 -pacbio-corrected ATCC20_CCS.fastq useGrid=remote gnuplot="~/software/anaconda3/envs/python2/bin/gnuplot" java="~/software/anaconda3/envs/python2/bin/java" minThreads=10 maxThreads=60

G. Bin quality evaluation

checkm lineage_wf -t 10 -x fasta bin_folder output_folder > output_file

H. Contig binning

perl run_MaxBin.pl -contig contig.fasta -reads read1.fastq.gz -reads2 read2.fastq.gz -out output_dir -threads 32

I. Calculate basic statistics

quast -o output_dir input.fasta

J. tRNA prediction

aragorn input.fasta -o result.txt

K. rRNA prediction

barrnap –quiet input.fasta > result.txt

L. annotate taxonomic classification

kraken --threads 8 --db kraken_db --output result.txt input.fasta

kraken-report --db kraken_db result.txt > result.report
